# Mucosal BCG Vaccination Induces Protective Lung-Resident Memory T Cell Populations against Tuberculosis

**DOI:** 10.1128/mBio.01686-16

**Published:** 2016-11-22

**Authors:** Carolina Perdomo, Ulrike Zedler, Anja A. Kühl, Laura Lozza, Philippe Saikali, Leif E. Sander, Alexis Vogelzang, Stefan H. E. Kaufmann, Andreas Kupz

**Affiliations:** a Department of Immunology, Max Planck Institute for Infection Biology, Berlin, Germany; b Department of Medicine, Charité University Hospital, Berlin, Germany; c Department of Infectious Diseases and Pulmonary Medicine, Charité University Hospital, Berlin, Germany; d Centre for Biosecurity and Tropical Infectious Diseases, Australian Institute of Tropical Health and Medicine, James Cook University, Cairns, Queensland, Australia

## Abstract

*Mycobacterium bovis* Bacille Calmette-Guérin (BCG) is the only licensed vaccine against tuberculosis (TB), yet its moderate efficacy against pulmonary TB calls for improved vaccination strategies. Mucosal BCG vaccination generates superior protection against TB in animal models; however, the mechanisms of protection remain elusive. Tissue-resident memory T (T_RM_) cells have been implicated in protective immune responses against viral infections, but the role of T_RM_ cells following mycobacterial infection is unknown. Using a mouse model of TB, we compared protection and lung cellular infiltrates of parenteral and mucosal BCG vaccination. Adoptive transfer and gene expression analyses of lung airway cells were performed to determine the protective capacities and phenotypes of different memory T cell subsets. In comparison to subcutaneous vaccination, intratracheal and intranasal BCG vaccination generated T effector memory and T_RM_ cells in the lung, as defined by surface marker phenotype. Adoptive mucosal transfer of these airway-resident memory T cells into naive mice mediated protection against TB. Whereas airway-resident memory CD4^+^ T cells displayed a mixture of effector and regulatory phenotype, airway-resident memory CD8^+^ T cells displayed prototypical T_RM_ features. Our data demonstrate a key role for mucosal vaccination-induced airway-resident T cells in the host defense against pulmonary TB. These results have direct implications for the design of refined vaccination strategies.

## IMPORTANCE

BCG remains the only licensed vaccine against TB. Parenterally administered BCG has variable efficacy against pulmonary TB, and thus, improved prevention strategies and a more refined understanding of correlates of vaccine protection are required. Induction of memory T cells has been shown to be essential for protective TB vaccines. Mimicking the natural infection route by mucosal vaccination has been known to generate superior protection against TB in animal models; however, the mechanisms of protection have remained elusive. Here we performed an in-depth analysis to dissect the immunological mechanisms associated with superior mucosal protection in the mouse model of TB. We found that mucosal, and not subcutaneous, BCG vaccination generates lung-resident memory T cell populations that confer protection against pulmonary TB. We establish a comprehensive phenotypic characterization of these populations, providing a framework for future vaccine development.

## INTRODUCTION

Since its introduction almost a century ago (1), live attenuated *Mycobacterium bovis* Bacille Calmette-Guérin (BCG) remains the only licensed vaccine against tuberculosis (TB) caused by the intracellular pathogen *Mycobacterium tuberculosis*. Although originally applied orally, today BCG is administered intradermally in early childhood and effectively prevents extrapulmonary TB, mainly disseminated miliary and meningeal forms in children (2). However, BCG fails to confer sufficient protection against the most common form of the disease, pulmonary TB. Thus, TB continues to cause significant global morbidity and mortality (3). The development and implementation of new and more efficient vaccines is mandatory if TB morbidity and mortality are to be reduced by 90 and 95%, respectively, to achieve the 2035 goal of the Stop TB Partnership (4, 5).

Induction of memory T cells has been shown to be essential for protective TB vaccines (6). In mice, protection against an *M. tuberculosis* challenge following subcutaneous (s.c.) BCG vaccination is dependent on T helper type 1 (Th1) CD4^+^ T cell responses (7, 8). However, one of the shortcomings of s.c. BCG administration is the overall weak memory lymphocyte generation, which in addition lacks the mucosal-homing chemokine receptors that allow migration to the lung (9). Hence, mucosal vaccination has been suggested as a mimic of natural infection in order to improve local immunity at the site of infection (10–12). Comprehensive analyses of local immunity and correlates of protection in both the lung airways and the parenchyma are essential for the rational design of mucosal TB vaccination strategies using BCG (13, 14). Airway luminal T cells have been found to be critical for protection against TB (15). However, in-depth characterization of infiltrating antigen-specific immune cell populations, in particular localization and function of tissue resident memory T (T_RM_) cell subsets generated by mucosal vaccination, is still lacking.

Until recently, memory T cells were subdivided into two main subsets (16). First, T cells expressing high levels of CD62L, termed central memory T (T_CM_) cells, migrate to lymphoid organs in response to l-selectin ligands, and second, low levels of CD62L mark T effector memory T (T_EM_) cells, which recirculate between blood and peripheral tissues, where they are thought to survey the initial portals of infection (17). More recently, a third subset of memory T cells, T_RM_ cells, which permanently resides in nonlymphoid tissues, has been mostly described (18) as CD69^+^ CD103^+^. Because of their strategic location and rapid recall response, T_RM_ cells represent preferred cellular targets for efficacious vaccination. Whether mucosal BCG vaccination generates protective T_RM_ cells in the lung remains to be explored. Our study investigated the hypothesis that an accumulation of *Mycobacterium*-specific lung-resident T cells, some of them expressing the T_RM_ phenotype, underpins the improved protection against TB seen following the mucosal administration of BCG.

## RESULTS

### Mucosal BCG vaccination confers superior protection against *M. tuberculosis* infection.

**FIG 1:**
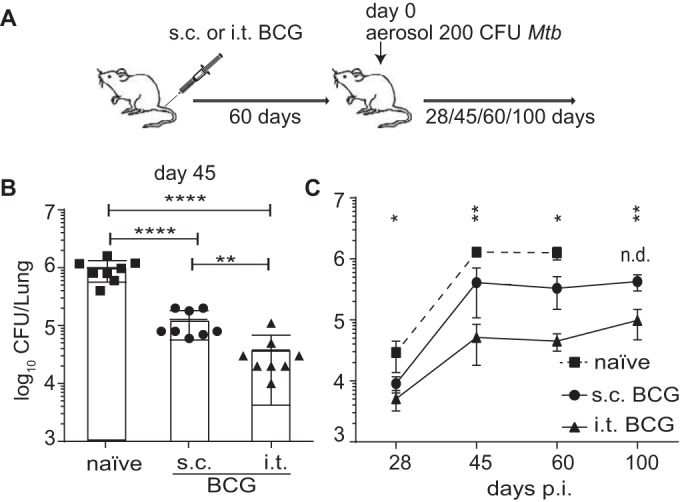
Mucosal BCG vaccination confers improved protection against *M. tuberculosis* infection. (A) B6 mice were BCG vaccinated either i.t. or s.c. Sixty days later, vaccinated and control groups were aerosol infected with a low dose of virulent *M. tuberculosis* and the CFU counts in their lungs were determined at the time points indicated. (B, C) Individual log_10_ CFU counts per lung (48) at day 45 p.i. (B) and mean log_10_ CFU counts per lung from two pooled independent experiments ± the standard error of the mean at the time points indicated (*n* = 8 mice per group) (C). The statistical significance of differences between the s.c. and i.t. BCG vaccination routes is shown. ****, *P* ≤ 0.0001; **, *P* ≤ 0.01; *, *P* ≤ 0.05; n.d., not done (analysis of variance with Tukey’s posttest for significance).

To investigate the role of lung-resident T cells in immune protection against TB following BCG vaccination, we compared local (mucosal) BCG vaccination via the intratracheal (i.t.) route to parenteral vaccination by s.c. administration of BCG. Sixty days after vaccination, mice were challenged aerogenically with *M. tuberculosis* and the bacterial loads in their lungs were determined at various time points postinfection (p.i.) (Fig. 1A). Confirming recent studies (19, 20), we found that mucosal BCG vaccination confers better protection against *M. tuberculosis* infection than parenteral s.c. BCG vaccination for at least 100 days (Fig. 1B and C).

### Mucosal BCG vaccination generates a transient influx of *Mycobacterium*-specific CD4^+^ and CD8^+^ T cells into the lung parenchyma.

**FIG 2:**
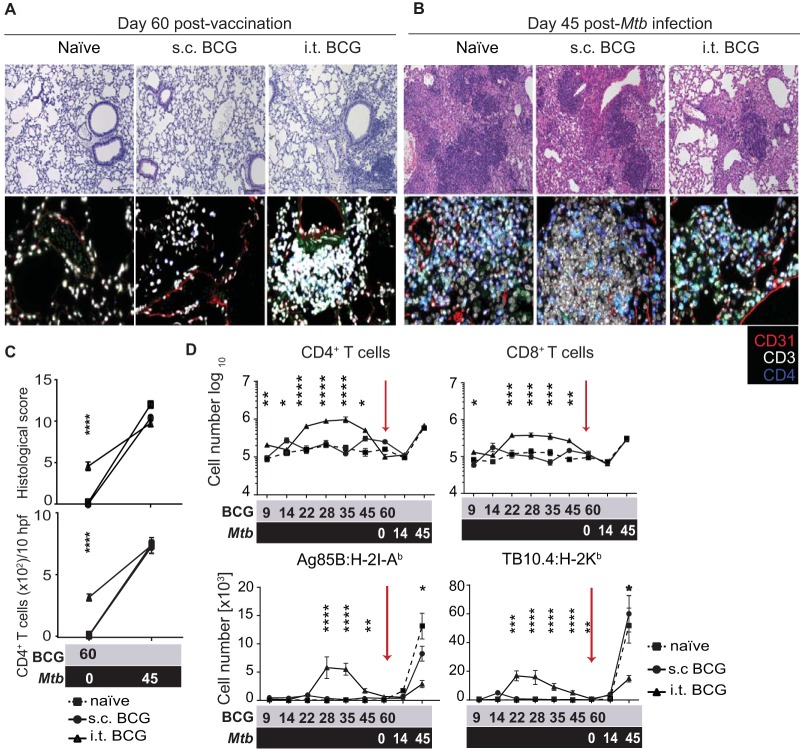
I.t. BCG vaccination causes transient influx of T cells into the lung parenchyma. Histological staining of lung sections from control and i.t. or s.c. BCG-vaccinated mice 60 days after BCG immunization (A) and on day 45 after *M. tuberculosis* infection (B). Lung sections were stained with H&E (top) and IF (bottom) for CD31 (red), CD3 (white), and CD4 (blue). (C) Histological scores (top) and numbers of CD4^+^ T cells per 10 high-power fields (hpf) (bottom) at designated time points after BCG vaccination (gray) and an *M. tuberculosis* challenge (black). Scale bar, 100 μm. Flow cytometric quantification of lung parenchyma (D) TCRβ^+^ CD4^+^ and CD8^+^ T cells (top) and antigen-specific Ag85B:H-2I-A^b^ CD4^+^ and TB10.4:H-2K^b^ CD8^+^ T cells (bottom) at designated time points after BCG vaccination (gray) and an *M. tuberculosis* challenge (black). Results are presented as mean values ± the standard error of the mean from two pooled independent experiments (*n* = 8 to 10 mice per group). The statistical significance of differences between s.c. and i.t. BCG immunizations is shown. ****, *P* ≤ 0.0001; ***, *P* ≤ 0.001; **, *P* ≤ 0.01; *, *P* ≤ 0.05. (C, D) Analysis of variance with Tukey’s posttest for significance.

To identify possible mechanisms of improved protection following i.t. BCG vaccination, we performed a histological analysis of lung-infiltrating immune cells. Sixty days after mucosal vaccination (immediately prior to infection), unperfused lungs displayed greater cell infiltration and higher histological scores than those of naive and s.c. BCG-vaccinated mice (Fig. 2A and C, top). A large proportion of lung-infiltrating cells were CD3^+^ T cells, many of which were CD4^+^ (Fig. 2A and C, bottom). In contrast, 45 days after *M. tuberculosis* infection, there were no significant differences in the total number of T cells among the groups despite the lower histological scores of BCG-vaccinated animals (Fig. 2B and C).

To determine whether lung-infiltrating T cells were located in the lung parenchyma or the lung airways, we first removed the bronchoalveolar lavage fluid (BALF) and performed flow cytometry of the lung parenchyma tissue. Mucosal BCG vaccination induced higher numbers of CD4^+^ and CD8^+^ T cells in the lung parenchyma between days 22 and 45 following BCG vaccination (Fig. 2D, top). Intriguingly, this increase proved to be transient, as by day 60, the day of an *M. tuberculosis* challenge, there were no significant differences in the total lung parenchyma-infiltrating T cell numbers between the vaccination routes (Fig. 2D, top). At that time point, approximately 100 BCG CFU were detected in the lung (see Fig. S1A in the supplemental material). The majority of lung-parenchyma-infiltrating T cells displayed a memory phenotype, and a proportion stained positive for major histocompatibility complex (MHC) peptide tetramers derived from dominant mycobacterial antigens, namely, Ag85B-specific CD4^+^ (Ag85B:H-2I-A^b^) and TB10.4-specific CD8^+^ (TB10.4:H-2K^b^) T cell subpopulations (Fig. 2D, bottom; see Fig. S1B). However, apart from a small number of persisting TB10.4^+^-specific CD8^+^ T cells, the overall numbers of antigen-specific CD4^+^ and CD8^+^ T cells were comparable between the i.t. and s.c. BCG-vaccinated groups directly before an *M. tuberculosis* challenge (Fig. 2D, bottom) (21). Furthermore, no significant differences in the numbers of lung alveolar macrophages (AMs) (CD11c^hi^ CD11b^lo^ F4/80^+^), dendritic cells (DCs) (CD11c^hi^ CD11b^lo^ F4/80^lo^ MHC class II^hi^) or neutrophils (CD11b^hi^ Ly6G^hi^) were observed over time between the two routes of vaccination, which suggests that changes in the myeloid compartment did not underlie increased protection (see Fig. S1C). Collectively, these results suggest that mucosal BCG vaccination drives a transient increase in *Mycobacterium*-specific CD4^+^ and CD8^+^ T cell populations in the lung parenchyma that recedes before a challenge.

### I.t. BCG vaccination generates T cells seeding the lung airways.

**FIG 3:**
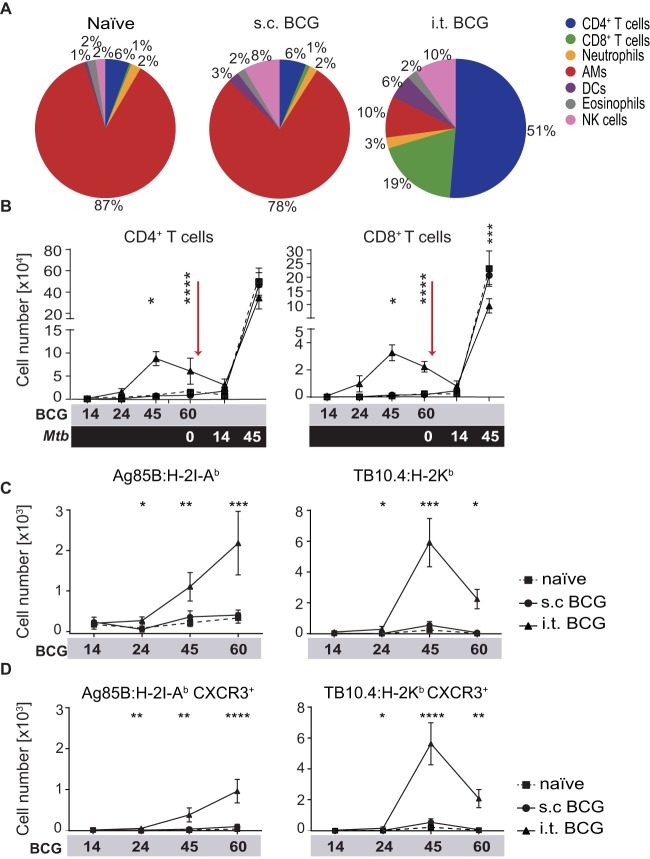
I.t. BCG vaccination generates T cells seeding the lung airways. (A) Pie charts illustrating the composition of BALF cell populations as proportions of the total leukocytes from naive mice and mice at 60 days after BCG vaccination. (B) Flow cytometric quantification of total TCRβ^+^ CD4^+^ and CD8^+^ T cells in BALF at the time points indicated after BCG vaccination (gray) and an *M. tuberculosis* challenge (black). (C, D) Quantification of total BALF TCRβ^+^ Ag85B:H-2I-A^b^ CD4^+^ and TB10.4:H-2K^b^ CD8^+^ T cells (C) and TCRβ^+^ Ag85B:H-2I-A^b^ CD4^+^ and TB10.4:H-2K^b^ CD8^+^ T cells expressing CXCR3 (D) at designated time points after BCG vaccination. Results are presented as pooled mean data ± the standard error of the mean (B to D) or representative images (A) from two pooled independent experiments (*n* = 6 to 8 mice per group). The statistical significance of differences between s.c. and i.t. BCG vaccinations is shown. ****, *P* ≤ 0.0001; ***, *P* ≤ 0.001; **, *P* ≤ 0.01; *, *P* ≤ 0.05. (B to D) Analysis of variance with Tukey’s posttest for significance.

To further determine the contribution of airway-resident immune cells to improved vaccine-mediated protection, we collected BALF and performed a comprehensive analysis of airway-infiltrating cells in response to BCG vaccination and *M. tuberculosis* challenge. Analysis of the proportional changes in lumen-resident cell types revealed a cellular composition skewed toward resident lymphocytes following mucosal vaccination (Fig. 3A; see Fig. S2A). Although the total cell numbers in the BALF were comparable (see Fig. S2B), increased and decreased frequencies in airway cell populations were also reflected in the total cell numbers (see Fig. S2C). In contrast to the kinetics of local parenchymal T cell populations, we identified increased frequencies and numbers of airway luminal T cells after i.t. BCG vaccination that persisted until the challenge (Fig. 3B). Influx of T cells into the lung airways was detected at later experimental time points than parenchymal infiltration and started around day 24 after vaccination. Most strikingly, i.t. BCG vaccination led to a profound change in the composition of lung-residing immune cells that was characterized by a numerical and proportional increase in T cells (Fig. 3B; see Fig. S2A), many of which were specific for mycobacterial antigens by tetramer staining (Fig. 3C; see Fig. S3). Additionally, CXCR3, a chemokine receptor required for migration of T cells into the lung airways and parenchyma (22), was highly expressed on antigen-specific T cells after i.t. BCG vaccination (Fig. 3D), indicating recent targeted migration to the lung airways.

### T_EM_ and T_RM_ cells infiltrate the lung airways after i.t. BCG vaccination.

**FIG 4:**
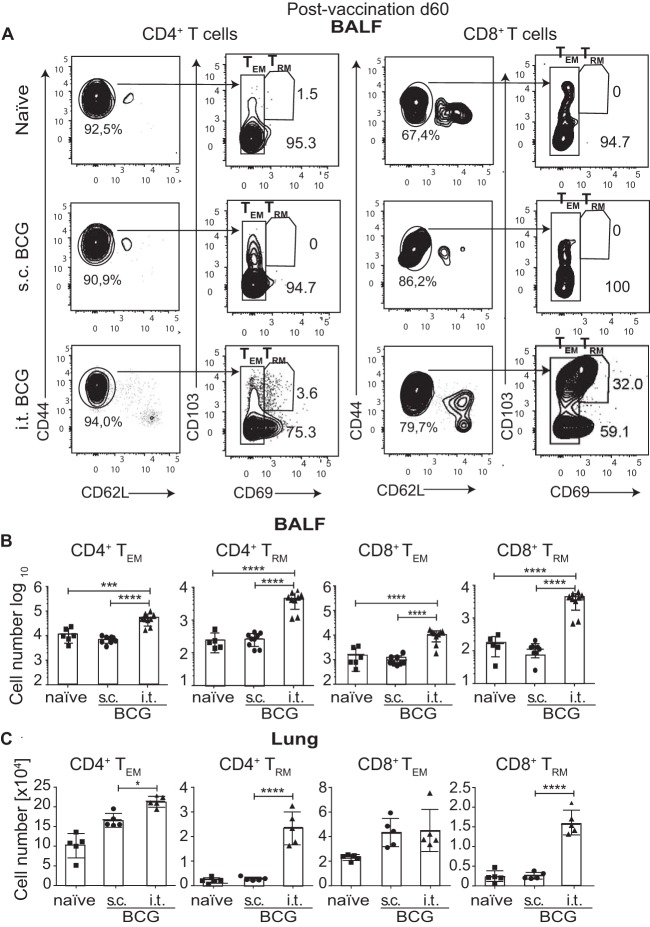
T_EM_ and T_RM_ cells infiltrate the lung airways after i.t. BCG vaccination. (A) Representative flow cytometry gating strategy for T_EM_ and T_RM_ cells among CD4^+^ and CD8^+^ T cells at day 60 after BCG immunization in BALF pregated on TCRβ^+^ CD4^+^ or TCRβ^+^ CD8^+^ T cells. (B, C) Quantification of total CD4^+^ T_EM_, CD4^+^ T_RM_, CD8^+^ T_EM_, and CD8^+^ T_RM_ cells by flow cytometry 60 days after BCG vaccination in BALF (B) and lung parenchyma (C). Results are presented as pooled mean data ± the standard error of the mean plus individual data points (B, C) or representative fluorescence-activated cell sorter plots (A) from two pooled independent experiments (*n* = 5 to 10 mice per group). ****, *P* ≤ 0.0001; ***, *P* ≤ 0.001; *, *P* ≤ 0.05. (B, C) Analysis of variance with Tukey’s posttest for significance.

Because of the striking increase in the number of luminal T cells following i.t. vaccination, we interrogated whether airway-infiltrating T cells following i.t. BCG administration phenotypically resembled T_EM_ (CD44^hi^ CD62L^lo^ CD69^lo^) and T_RM_ (CD44^hi^ CD62L^lo^ CD103^+^ CD69^+^) cells. Particularly the T_RM_ population has been shown to confer protection against viral and bacterial pulmonary infections (23, 24). We found that, indeed, i.t. BCG vaccination recruited significantly higher frequencies and absolute numbers of CD4^+^ and CD8^+^ T_RM_ and T_EM_ cells to the airways than s.c. BCG vaccination (Fig. 4A and B). Similarly, characterization of parenchymal T cells revealed higher numbers of CD4^+^ and CD8^+^ T_EM_ and T_RM_ cells in i.t. BCG-vaccinated mice (Fig. 4C). Collectively, our results demonstrate that i.t. BCG vaccination induces CD4^+^ and CD8^+^ T_EM_ and T_RM_ cell recruitment to the lung airway spaces and the lung parenchyma.

### Phenotypic characterization of airway-infiltrating T cells generated by i.t. BCG vaccination.

**FIG 5:**
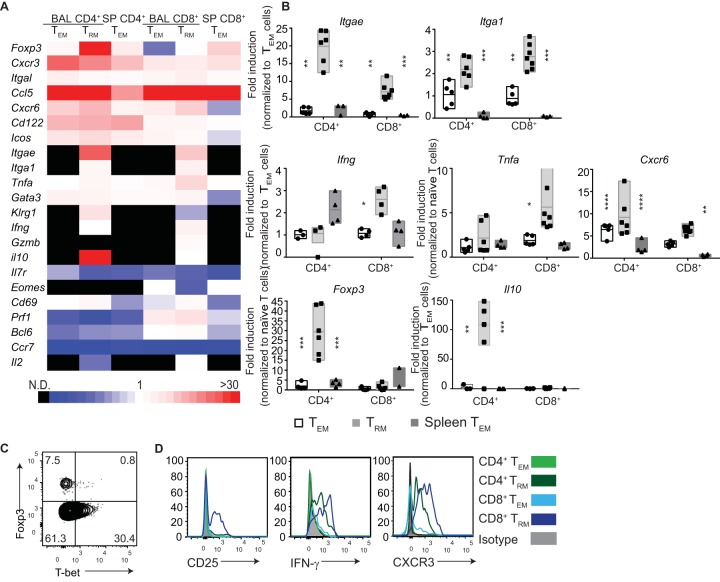
Phenotypic characterization of lung-infiltrating T cells generated by i.t. BCG vaccination (A, B). B6 mice were BCG vaccinated i.t., and BALF T cell subsets were sorted 60 days later by fluorescence-activated cell sorting gated as described in the legend to Fig. 4A. Sorted naive (TCRβ^+^ CD44^lo^ CD62L^hi^) BALF T cells or splenic T_EM_ cells (TCRβ^+^ CD44^hi^ CD62L^lo^) from i.t. BCG-vaccinated mice 60 days after immunization were also used as controls. (A) Heat map showing gene expression from sorted BALF T cell populations. Triplicates of 100 BALF CD4^+^ and CD8^+^ T_EM_ and T_RM_ cells from i.t. BCG-vaccinated mice were sorted. Quantitative PCR was run with the data collection software (36 cycles) from Fluidigm. mRNA concentrations of all sorted T cell populations were normalized to β-actin (NM_007393.4) expression. The color code indicates fold changes (2^−ΔΔ*CT*^) in transcripts relative to the appropriate internal control as indicated. (B) Fold changes in the expression of selected genes of sorted BALF CD4^+^ and CD8^+^ T_EM_ and T_RM_ cells from i.t. BCG-vaccinated mice compared to the appropriate internal control. Quantitative PCR was run with the data collection software (36 cycles) from Fluidigm as described for panel A. (C, D) BALF immune cell phenotype measured by flow cytometry 60 days after i.t. BCG vaccination. (C) Representative flow cytometry of intracellular Foxp3 and T-bet expression by sorted CD4^+^ T_RM_ cells. (D) Representative histograms of selected surface activation markers and IFN-γ expression by CD4^+^ and CD8^+^ T_EM_ and T_RM_ cells. Results are presented as pooled individual data points ± the standard error of the mean (B), representative fluorescence-activated cell sorter plots (C), or histograms (D) from two pooled independent experiments (*n* = 6 to 8 mice per group). The statistical significance of differences from the T_RM_ cell subset (B) is shown. ****, *P* ≤ 0.0001; ***, *P* ≤ 0.001; **, *P* ≤ 0.01; *, *P* ≤ 0.05. (B) Analysis of variance with Tukey’s posttest for significance.

T_RM_ cells vary in phenotype and function, depending on the tissue they reside in (25–27). The phenotype of T_RM_ cells in lung airways following mucosal BCG vaccination has not been characterized. Hence, we performed transcriptional gene expression profiling of sorted BALF CD4^+^ and CD8^+^ T_EM_ and T_RM_ cell subpopulations induced by i.t. BCG vaccination with a Fluidigm Dynamic Array. The purity of the different sorted cell populations was routinely assessed at 86 to 99% (see Fig. S4). Increased transcription levels of typical markers associated with tissue residency of CD4^+^ and CD8^+^ T_RM_ such as *Itgae* (CD103) and *Itga1* (VLA-1) were confirmed (Fig. 5A and B). CD4^+^ T_RM_ cells displayed a regulatory profile, with high *Foxp3* and *Il10* mRNA expression (Fig. 5A and B). Additionally, CD4^+^ T_RM_ cells expressed T-bet, as well as Foxp3, at the protein level (Fig. 5C). Importantly, each marker was expressed by distinct subpopulations, suggesting a heterogeneous population comprising effector and regulatory T cells (28). Therefore, we concluded that CD4^+^ T_RM_ cells, defined here as CD4^+^ CD103^+^ CD69^+^ cells, comprise a mixture of regulatory and effector T cells rather than solely belonging to the T_RM_ subset. On the other hand, CD8^+^ T_RM_ cells expressed significantly higher levels of gamma interferon (IFN-γ) (*Ifng*), tumor necrosis factor alpha (TNF-α) (*Tnfa*), and *Cxcr6* (Fig. 5B) (29) and statistically insignificantly higher levels of perforin (*Prf1*) and granzyme B (*Gzmb*) than their CD8^+^ T_EM_ counterparts (Fig. 5A).

To further characterize the phenotypes of T_EM_ and T_RM_ cells infiltrating the airways after i.t. BCG vaccination, we also assessed interleukin-2 (IL-2) receptor alpha chain (CD25), IFN-γ, and CXCR3 protein expression levels. I.t. BCG vaccination generated CD25- and CXCR3-expressing, IFN-γ-producing CD8^+^ T_RM_ cells, as well as CXCR3^+^-expressing, IFN-γ-producing CD4^+^ airway-resident T cell subpopulations (Fig. 5D). Collectively, these data indicate that i.t. BCG vaccination induced airway-resident T cells with a heightened ability to migrate to the lung and produce the key protective proinflammatory cytokine IFN-γ. Although CD4^+^ T cells could be categorized as T_EM_ and T_RM_ on the basis of surface markers, transcriptional profiling revealed more heterogeneous populations.

### Mucosal transfer of airway-resident T cell populations confers protection against TB.

**FIG 6:**
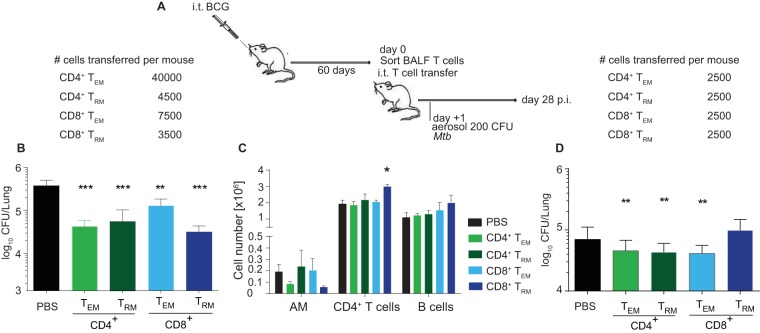
Mucosal transfer of airway-resident T cell populations confers protection against TB. (A) B6 mice were BCG vaccinated i.t., and BALF T cell subsets were sorted 60 days later by fluorescence-activated cell sorting. BALF CD4^+^ and CD8^+^ T_EM_ and T_RM_ cells were sorted as depicted in Fig. 4A. The sorted T cell population purity was assessed as >86%. From 0.25 × 10^4^ to 4 × 10^4^ sorted cells were i.t. transferred into naive B6 mice (B, D). The numbers of cells transferred are indicated at the top. The following day, recipient mice were aerosol infected with *M. tuberculosis* and lung CFU counts were determined 28 days later. (B, D) Bacterial CFU counts in lung tissue after BALF removal (B) and in airways and tissue without lavage (D). (C) Immune cellular composition in the lung parenchyma 28 days p.i following i.t. transfer of sorted BALF T cell populations from i.t. BCG-vaccinated mice. Cell numbers are representative of one of two experiments performed as described for panel B. The statistical significance of differences from the i.t. PBS control is shown. Results are presented as mean pooled data ± the standard error of the mean (B to D) from one representative (B, C) or two pooled independent experiments (D) (*n* = 3 mice per group [B, C] or *n* = 6 to 8 mice per group [D]). ****, *P* ≤ 0.0001; ***, *P* ≤ 0.001; **, *P* ≤ 0.01; *, *P* ≤ 0.05. (B to D) Analysis of variance with Tukey’s posttest for significance.

To determine the subset(s) of airway-infiltrating T cells critical for improved protection after mucosal vaccination, we adoptively transferred sorted airway T cell subpopulations directly into the tracheas of naive C57BL/6 (B6) mice 1 day prior to an aerogenic *M. tuberculosis* challenge (Fig. 6A; see Fig. S4 in the supplemental material). All of the transferred subsets provided some degree of protection 28 days after the *M. tuberculosis* challenge (Fig. 6B). Intriguingly, transfer of as few as 3,500 sorted CD8^+^ T_RM_ cells into naive mice conferred the most profound protection against a *M. tuberculosis* challenge, on a per-cell basis (Fig. 6B). Transfer of CD8^+^ T_RM_ cells was associated with significantly lower AM numbers, higher numbers of CD4^+^ T cells, and increased numbers of B cells in the lung 28 days after the *M. tuberculosis* challenge (Fig. 6C). We also performed airway CD4^+^ and CD8^+^ T cell depletion (Fig. S5; see Fig. S6). These experiments yielded an opposite effect compared to the transfer of different T cell subsets (Fig. 6C). Whereas the mucosal CD4^+^ T cell depletion efficiency was around 90%, the CD8^+^ T cell depletion efficiency was only around 50% (data not shown). Because of the low efficiency of CD8^+^ T cell depletion, we could not draw any definitive conclusions. Therefore, despite its great additive value to the overall conclusion, we were not able to specifically delete T_RM_ cell populations from the airway.

Intriguingly, when the bacterial load in the whole lung was determined without previously performing lavage, the transfer of all airway T cell subsets reduced bacterial loads at equal levels and the improved protective effect of CD8^+^ T_RM_ cells was lost (Fig. 6D). These results suggest that i.t. BCG vaccination induces (i) multiple subpopulations of local T_RM_ cells that contribute to protection against *M. tuberculosis* and (ii) compartmentalized protective effects in lung airways but not in lung parenchyma.

### Oral and i.n. vaccinations mimic i.t. BCG vaccination.

**FIG 7:**
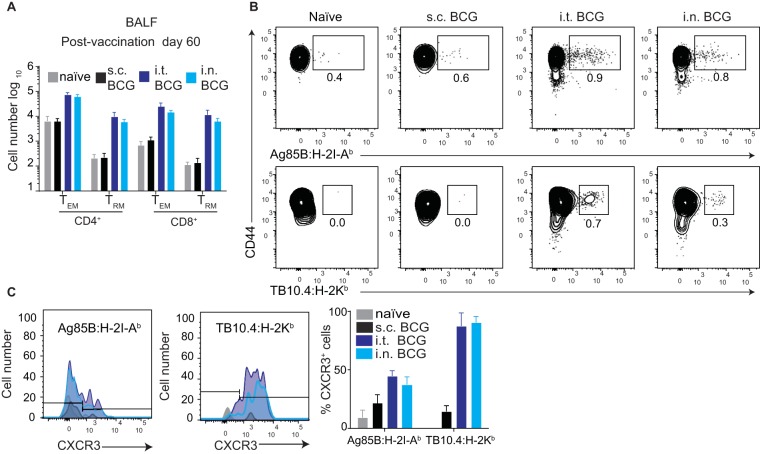
I.n. BCG vaccination mimics i.t. BCG vaccination. (A to C) BALF characterization at day 60 after BCG vaccination. (A) Quantification of BALF TCRβ^+^ CD4^+^ and CD8^+^ T cell and T_EM_ and T_RM_ cell numbers by flow cytometry. (B) Flow cytometry gating strategy for Ag85B:H-2I-A^b^ CD4^+^ and TB10.4:H-2K^b^ CD8^+^ T cells pregated on TCRβ^+^ CD4^+^ or CD8^+^ T cells. (C) Representative histogram showing CXCR3 expression by Ag85B:H-2I-A^b^ CD4^+^ and TB10.4:H-2K^b^ CD8^+^ T cells (left) and their percentage of expression by Ag85B:H-2I-A^b^ CD4^+^ and TB10.4:H-2K^b^ CD8^+^ T cells following BCG vaccination (49). Results are presented as pooled mean data ± the standard error of the mean (A, C), as representative fluorescence-activated cell sorter plots (B), or as histograms (C) from two pooled independent experiments (*n* = 8).

Finally, although the i.t. BCG administration employed in our model is a low-invasion intervention, it is unlikely to be broadly applicable as a human vaccination strategy. Clinically more feasible intranasal (i.n.) and oral BCG vaccinations strikingly induced infiltration of T cells into the lung parenchyma and airways very similar to that induced by i.t. BCG vaccination, which was reflected in overall increased numbers of T_EM_ and T_RM_ cells (Fig. 7A), as well as *Mycobacterium*-specific T cells expressing CXCR3 (Fig. 7B and C; see Fig. S7). Together with published observations regarding improved *M. tuberculosis* control following i.n. and oral BCG vaccinations (30, 31), our data indicate that mucosal BCG vaccination promotes protection via potent induction of lung parenchyma- and airway-resident memory CD4^+^ and CD8^+^ T cells, comprising mixed CD4^+^ T cell populations and CD8^+^ T_RM_ cells.

## DISCUSSION

We describe an in-depth *in vivo* approach to dissection of the immunological mechanisms associated with improved protection of mucosal BCG vaccination against pulmonary TB. We conclude that lung-resident CD4^+^ and CD8^+^ T cells, comprising CD8^+^ T_RM_ cells, are a main component underlying the enhanced efficacy of mucosal BCG administration. Airway-resident CD4^+^ T cells comprised a mixture of T-bet^+^ effector and Foxp3^+^-expressing regulatory T cells. In contrast, airway-resident CD8^+^ T cells displayed prototypical T_RM_ features and expressed IFN-γ and TNF-α, two major mediators of protective immunity against *M. tuberculosis*.

It has been previously shown that transfer of total lung T cells following i.n. but not s.c. vaccination with *M. tuberculosis* culture filtrate proteins can protect against TB (15). Aerosol administration of an attenuated *M. tuberculosis* vaccine candidate, *M. tuberculosis* Δ*sigH*, has also been reported to be highly effective in preventing TB in nonhuman primates via induction of local T cell responses (19). These findings validate the superiority of mucosal vaccination in generating a robust and effective T cell response against *M. tuberculosis*. As the most striking effect of i.t. BCG vaccination we identified a prominent subpopulation of CD8^+^ T_RM_ cells in the lung airways bearing the prototypic CD69^+^ CD103^+^ surface phenotype associated with tissue sequestration (32, 33). Many coexpressed the mucosal and lung-homing markers CD103 (*Itgae*) and VLA-1 (*Itga1*). CD69, an early leukocyte activation marker, can interact with S1P1 and downregulate its expression, leading to prolonged T cell retention and local memory formation (34). CD103 on T cells binds to epithelial E-cadherin in diverse organs such as the skin and gut (27). Our finding that CD103 is surface expressed, especially by CD8^+^ T_RM_ cells in the lung following mycobacterial lung infection, extends its relevance to lung-residing memory T cell responses. VLA-1, α1β1-integrin, is an adhesion molecule known to be highly expressed by respiratory virus-specific memory CD8^+^ T cells in the airways, retaining them in the lung through attachment to the extracellular matrix (35). Our study is the first to ascribe protective relevance to intraluminal T cells following mucosal BCG vaccination, which includes CD8^+^ T_RM_ cells in the lung airways in the context of TB.

CD8^+^ T_RM_ cells have been implicated in protection following viral infections (36), but their beneficial role following bacterial infection is just being appreciated (37). Recent work has highlighted the potential of CD8^+^ T_RM_ to activate bystander NK and B cells via IFN-γ, TNF-α, and IL-2, in addition to their well-known cytolytic role (24). Mucosal i.t. transfer of airway T cell populations into naive mice identified a crucial role for CD8^+^ T_RM_ cells in conferring lung protection in our study. Intriguingly, when BALF was collected from infected mice prior to CFU enumeration, transferred CD8^+^ T_RM_ not only displayed superior protection against an *M. tuberculosis* challenge but also reduced the number of AMs and increased the local CD4^+^ T and B cell numbers. In contrast, when CFU counts in the complete lung (BALF plus lung tissue) were determined, the protective capacity of transferred CD8^+^ T_RM_ cells was lower. Thus, it is tempting to speculate that cytolytic CD8^+^ T_RM_ cells limit the entry of *M. tuberculosis* into lung tissue by killing infected AMs in the lung airways, constraining the cellular reservoir for *M. tuberculosis*. It is also possible that CD8^+^ T_RM_ cells contribute to protective immunity attained by i.t. BCG vaccination in the lung (i) by means of killing infected AMs and (ii) by recruiting CD4^+^ T cells to the site of *M. tuberculosis* infection. CD8^+^ T_RM_ cells’ killing abilities, as well as their compartmentalized protective role, should be addressed in future studies.

Comprehensive transcriptional and flow cytometric analysis of airway CD4^+^ memory T cells identified a heterogeneous population comprising Foxp3^+^- or T-bet^+^-expressing T cell subsets. Further studies are needed to analyze the functional properties of CD103^−^ CD69^+^ CD4^+^ memory T cells that have been described by others as most concordant to the CD4^+^ T_RM_ population (38). In addition, enhanced IL-10 transcripts suggest diverse roles for lung CD4^+^ T cells besides the classical Th1 responses previously considered correlates of protection. Although it was beyond the scope of this study to dissect the underlying protective mechanism, CD4^+^ Foxp3^+^ T-cell-derived IL-10 emerges as a strong candidate for ameliorating immunopathology (39) and at the same time has been shown to promote the maturation of CD8^+^ T cells (40). The exact role of airway-resident CD4^+^ T-cell-derived IL-10 and its functional impact on local anti-*M. tuberculosis* immunity should be elucidated in future studies.

Further studies are required to dissect the mechanisms of protection induced by the transfer of total BALF. A minute number of influenza virus-specific CD8^+^ T cells in the airways was recently shown to be sufficient to transfer protection against a subsequent influenza virus infection (36). Therefore, it is possible that even fewer than the 2,500 lung-resident T cells induced by BCG vaccination that were transferred here could mediate protection after mucosal transfer. Although it is beyond the scope of this study, identifying the minimal number of T cells required to transfer protection will be valuable additional information.

Some remaining questions need to be addressed in future studies to determine the role of live antigen in the lung following mucosal BCG vaccination. Although the cellular analyses of the lung revealed similar results with s.c. and i.t. BCG-vaccinated mice, the presence of a low-grade ongoing infection in the lungs hampers the use of CD44 as a memory marker, as CD44 is also a marker of effector cells during ongoing infection. The crossover of CD69 as both an early activation marker and a resident memory marker requires further transfer experiments with T cell subpopulations to address their long-term viability and recall responses in the absence of antigen in order to validate them as “true” memory populations. Because there are no singular defining markers for T_RM_ cells yet, particularly for the CD4^+^ subset, in this study, we chose to perform mRNA phenotyping of CD4 and CD8 T cells infiltrating the airways postvaccination, which revealed heterogeneous expression of transcription factors and effector molecules and confirmed their ability to mount a recall response to an infectious challenge. Furthermore, it will also be important to determine the contribution of non-*M. tuberculosis*-specific memory T cells (e.g., influenza virus-specific T cells) in mediating protection after adoptive transfer. Although unspecific mechanisms for protection cannot be ruled out entirely, the fact that not all *M. tuberculosis*-specific T cell subsets protected equally well after adoptive transfer suggests that non-*M. tuberculosis*-specific effects (41) contribute little to protection against TB following vaccination. Nevertheless, only transfer of *M. tuberculosis*-unrelated memory T cell subsets from the lung will definitively address the role of noncognate effects.

Taken together, our results highlight the value of better understanding the mechanisms underlying mucosal vaccination against TB. Our findings emphasize that mucosal vaccination offers an option for improving protective efficacy against TB either by BCG or by second-generation vaccine candidates. We recommend that optimization of mucosal vaccine administration should complement the design of novel vaccine candidates that either substitute for or boost BCG immunization.

## MATERIALS AND METHODS

### Animals and bacteria.

B6 mice were maintained under specific-pathogen-free conditions. All experiments were conducted in accordance with the requirements of and approval by the State Office for Health and Social Services. *M. tuberculosis* strain H37Rv (ATCC no. 27294) and BCG SSI 1331 (ATCC no. 35733) were grown by previously described protocols (42). Prior to vaccination, vaccine stock vials were thawed and cells were harvested and resuspended in phosphate-buffered saline (PBS). For CFU enumeration, serial dilutions were performed and plated onto Middlebrook 7H11 agar. Plates were incubated at 37°C for 3 to 4 weeks prior to counting.

### Immunizations and infection.

B6 mice were immunized with 5 × 10^5^ (i.t. and i.n.), 1 × 10^6^ (s.c.), or 1 × 10^8^ (oral) CFU (12, 31, 43). For i.t. immunization, anesthetized mice (1:1:8 xylazine-ketamine-PBS) were inoculated in the oropharynx with 50 μl of bacteria (44). To determine protective efficacy, mice were challenged via the aerosol route with 200 CFU of *M. tuberculosis* H37Rv 60 days postvaccination by using a Glas-Col inhalation exposure system.

### Histology and IF assay.

Unperfused lungs from BCG-vaccinated or *M. tuberculosis*-infected animals were fixed for 24 h in 4% (wt/vol) paraformaldehyde and then dehydrated and embedded in paraffin for histological analysis. Two-micrometer sections were deparaffinized and stained with hematoxylin and eosin (H&E). For immunofluorescence (IF) assay, heat-induced antigen retrieval in citrate buffer (10 mM citric acid, 0.05% Tween 20, pH 6.0) was performed prior to incubation with anti-CD31 (clone SZ31; Dianova), anti-CD3 (clone M-20; Santa Cruz), and anti-CD4 (clone 4SM95; eBioscience) antibodies.

### Cell isolation.

Intra-airway luminal cells were removed from the lung by bronchial lavage as described previously (45). Supernatant was frozen at −80°C until protein analysis, and the remaining cells were analyzed by flow cytometry. Lungs were perfused with PBS through the left ventricle and cut into small pieces, and single-cell suspensions were prepared by mechanical dissociation through a 70-μm nylon mesh (46).

### Flow cytometry, intracellular cytokine staining, and tetramer staining.

Identification of innate cell populations was performed with antibodies against CD11b (M1/70), CD11c (HL3), Ly6G (1A8), Siglec-F (E50-2440), F4/80 (BM8), and MHC class II (M5/114.15.2). Surface identification of T cells was performed with antibodies against T cell receptor β (TCRβ) (H57-597), CD4 (GK1.5), CD8 (53-6.7), CD44 (IM7), CD62L (MEL-14), CD103 (M290), and CD69 (H1.2F3). For memory phenotyping, CXCR3 (CXCR3-173) and CD25 (PC61.5) antibodies were included. DCs were characterized as CD11c^hi^ MHC-II^hi^, AMs were characterized as CD11c^hi^ Siglec-F^hi^ CD11b^low-int^ autofluorescence positive, neutrophils were characterized as Ly6G^hi^ CD11b^hi^, eosinophils were characterized as CD11c^low/−^ Siglec-F^hi^, NK cells were characterized as TCRβ-NK1.1^+^, and CD4^+^ and CD8^+^ T cells were characterized as TCRβ^+^ CD4^+^ or TCRβ^+^ CD8^+^. Intracellular staining for transcription factors Foxp3 (FJK-16s) and T-bet (4B10) was performed with the Foxp3 staining buffer kit (eBioscience). To determine IFN-γ (4S.B3) cytokine levels, intracellular staining was performed with the BD Cytofix/Cytoperm Fixation/permeabilization kit according to manufacturer’s instructions.

Ag85B:H-2I-A^b^ (280 to 294: FQDAYNAAGGHNAVF) tetramers were provided by the National Institutes of Health tetramer facility, and TB10.4:H-2K^b^ (4 to 11: IMYNYPAM) tetramers were prepared in house. Tetramer staining was performed at room temperature for 1 h prior to additional surface staining. Analysis was performed on an LSR II or Canto II (Becton, Dickinson) flow cytometer. Data were analyzed with FlowJo (TreeStar).

### Mucosal T cell transfer.

CD4^+^ and CD8^+^ T_EM_ and T_RM_ cells were sorted from BALF collected from i.t. BCG-vaccinated mice 60 days postvaccination. B6 recipient mice were anesthetized and received 50 μl of a cell suspension in PBS containing sorted T cell populations i.t. The specific cell numbers transferred are indicated in the figures.

### Mucosal T cell depletion.

B6 mice were BCG vaccinated i.t., and 2 days prior to a challenge, CD4 and CD8 T cell subsets were mucosally depleted through i.t. administration of anti-CD4 (GK1.5), anti-CD8 (53-6.7), or anti-control IgG (Ctrl). Two days following mucosal depletion, depleted and untreated mice were aerosol infected with *M. tuberculosis* and lung CFU counts were determined 28 days later.

### Gene expression analysis.

Gene expression was analyzed simultaneously with the 48.48 Dynamic Array Integrated Fluidic Circuits from Fluidigm as previously described (47). Preamplification of genes by reverse transcription and cDNA synthesis (18 cycles) was performed with the Cells Direct one-Step qPCR kit (Life Technologies, Inc.) and TaqMan gene expression assay mix (Applied Biosystems). Triplicates of 100 BALF or lung parenchyma cells were sorted, and mRNA amounts were normalized to β-actin (NM_007393.4) expression. Data represent fold changes (2^−ΔΔ*CT*^) in transcripts relative to the appropriate internal control.

### Statistical analyses.

Statistical analyses were performed with GraphPad Prism software (San Diego, CA). For *in vivo* experiments, data from two independent experiments were pooled. *P* values of <0.05 were considered statistically significant.

## SUPPLEMENTAL MATERIAL

Figure S1  Lung immune cell infiltration following BCG vaccination. (A) Log_10_ CFU counts per lung at day 60 after i.t. or i.n. BCG administration. (B) Representative gating strategy for flow cytometric analysis of Ag85B:H-2I-A^b^ CD4^+^ and TB10.4:H-2K^b^ CD8^+^ T cells in lungs of naive and s.c. or i.t. BCG-vaccinated mice at day 60 after BCG immunization. T cells were pregated on TCRβ^+^ CD4^+^ or CD8^+^ T cells. (C) Total numbers of AMs, DCs, and neutrophils enumerated by flow cytometry at designated time points after BCG vaccination (gray) and after *M. tuberculosis* challenge (black). Results are presented as representative fluorescence-activated cell sorter plots from two pooled independent experiments (B), as individual data points (A), or as pooled mean data ± the standard error of the mean from two pooled independent experiments (C) (*n* = 5 to 10 mice per group). Statistical significance of differences between s.c. and i.t. BCG is shown. **, *P* ≤ 0.01; *, *P* ≤ 0.05 (analysis of variance with Tukey’s posttest for significance). Download Figure S1, EPS file, 1 MB

Figure S2  I.t. BCG vaccination leads to a profound change in the composition of lung airway-resident immune cells. (A) Representative gating strategy for flow cytometric analysis of total T cells, CD4^+^ and CD8^+^ T cells, AMs, and DCs in BALF 60 days after BCG vaccination. Flow cytometric quantification of total BALF cells (B) and innate-cell subsets (C) at day 60 after BCG vaccination. Results are presented as representative fluorescence-activated cell sorter plots (A) or as mean pooled data ± the standard error of the mean from two pooled independent experiments (B, C) (*n* = 8 to 10 mice per group). ****, *P* ≤ 0.0001; ***, *P* ≤ 0.001; *, *P* ≤ 0.05 (analysis of variance with Tukey’s posttest for significance). Download Figure S2, EPS file, 3.1 MB

Figure S3  I.t. BCG vaccination leads to infiltration of *Mycobacterium*-specific T cells to the airways. Representative gating strategy for flow cytometric analysis of Ag85B:H-2I-A^b^ CD4^+^ and TB10.4:H-2K^b^ CD8^+^ T cells in BALF 60 days after BCG vaccination. T cells were pregated on TCRβ^+^ CD4^+^ or CD8^+^ T cells. Results are presented as representative fluorescence-activated cell sorter plots from two pooled independent experiments (*n* = 8 to 10 mice per group). Download Figure S3, EPS file, 1 MB

Figure S4  Purity of sorted T cell populations used for adoptive cell transfer experiments and Fluidigm analysis. Representative gating strategy for flow cytometric analysis of transferred cells and gene expression profiling of cells, assessed by expression of CD103 and CD69 among TCRβ^+^ CD44^hi^ CD62L^lo^ CD4^+^ and CD8^+^ T cells in BALF 60 days after i.t. BCG vaccination. Download Figure S4, EPS file, 1.3 MB

Figure S5  Mucosal CD4 T cell depletion following i.t. BCG vaccination impairs protection against *M. tuberculosis* infection. (A) B6 mice were BCG vaccinated i.t., and 2 days prior to a challenge, CD4 and CD8 T cell subsets were mucosally depleted through i.t. administration of anti-CD4, anti-CD8, or anti-control IgG (Ctrl). Two days following mucosal depletion, depleted and untreated mice were aerosol infected with *M. tuberculosis* and lung CFU counts were determined 28 days later. (B) Lung bacterial CFU counts at day 28 after *M. tuberculosis* infection from two pooled independent experiments ± the standard error of the mean (*n* = 7 to 10 mice per group). **, *P* ≤ 0.01 (analysis of variance with Tukey’s posttest for significance). Download Figure S5, EPS file, 0.9 MB

Figure S6  Immune cell characterization after *M. tuberculosis* challenge following mucosal T cell depletion on i.t. BCG-vaccinated mice. (A) BALF total cell counts (left), frequency of lymphocytes (center), and total numbers of CD4 T cells, AMs, and B cells (49) at day 28 after *M. tuberculosis* infection. (B) Lung total cell counts (left) and total numbers of CD4 T cells, AMs, and B cells (49) at day 28 after *M. tuberculosis* infection. Results are presented as mean values ± the standard error of the mean from two pooled independent experiments (*n* = 5 to 10 mice per group). Unless specified otherwise, the statistical significance of differences from naive mice is shown. ***, *P* ≤ 0.001; **, *P* ≤ 0.01; *, *P* ≤ 0.05 (analysis of variance with Tukey’s posttest for significance). Download Figure S6, EPS file, 1.1 MB

Figure S7  Oral BCG vaccination mimics i.t. BCG vaccination. Quantification of total BALF TCRβ^+^ CD4^+^ and CD8^+^ T_EM_ and T_RM_ cell numbers 60 days after oral versus i.t. BCG vaccination. Results are presented as pooled mean data ± the standard error of the mean from two pooled independent experiments (*n* = 8). The statistical significance of differences between the oral and i.t. BCG vaccination routes is shown. ****, *P* ≤ 0.0001; *, *P* ≤ 0.05 (analysis of variance with Tukey’s posttest for significance). Download Figure S7, EPS file, 0.6 MB
